# Insanity as a Defence in the Criminal Courts—I

**Published:** 1908-10-10

**Authors:** 


### Medico-Legal Points.

### INSANITY AS A DEFENCE IN THE CRIMINAL COURTS—I.

The recent case at the Leeds Assizes, where a labourer was indicted for the murder of a woman whom he had killed and mutilated under particularly revolting circumstances, raises an interesting medico-legal question. At the outset the question was raised whether he was fit to plead. Dr. Edgerley, medical officer of the West Riding County Asylum, and Dr. Exley medical officer of Armley Gaol, were called to show that the prisoner was unfit to plead. They said that he suffered from insane delusions, which filled his mind, and largely impaired his faculty of attention. In answer to the judge, they conceded that he was able to understand what he was charged with, and the effect of the pleas of guilty and not guilty. His attention at any time could only be fixed by constantly addressing questions directly to him. The jury found that the prisoner was fit to plead, and in answer to the indictment he said "Guilty." Advised by the judge to alter his plea, he remarked, "Well, I am guilty. I did it. That is all I can say." He subsequently pleaded "not guilty." Mr. Justice Bigham directed the jury that the *onus* lay on the prisoner to satisfy them beyond all reasonable doubt that he did not know that he was doing wrong; if he knew he was doing wrong, it did not matter that he suffered from delusions, or hallucinations, or was "insane." A man commonly described as a lunatic may be as guilty of murder as anyone else. The jury found the prisoner guilty, and the judge expressed his approval of the verdict. The Court of Criminal Appeal found the man insane, and ordered him to be detained during his Majesty's pleasure.

All attempts at a short definition of the term insanity have proved unsatisfactory; perhaps the nearest approach to accuracy is obtained by thorough statement. That it is a chronic disease of the brain, inducing chronic disordered mental symptoms—the term disease being used in its widest acceptation. In the earliest epochs of medicine, the corporeal character of insanity was generally admitted, and it was not until the superstitious ignorance of the Middle Ages had obliterated the scientific, though by no means always accurate, deductions of the early writers that any theory of its purely psychical character arose. At the present day it is unnecessary to combat such a theory, as it is universally accepted that the brain is the organ through which mental phenomena are manifested, and therefore that it is impossible to conceive of the existence of an insane mind in a healthy brain. On this basis insanity may be defined as consisting in morbid conditions of the brain, the results of defective formation or altered nutrition of its substance induced by local or general morbid processes, and characterised especially by non-development, obliteration, impairment, or perversion of one or more of its psychical functions. Thus insanity is not a simple condition; it comprises a large number of diseased states of the brain, which have been gathered under one popular term on account of mental defect or aberration being the predominant symptom. The effect of insanity upon responsibility and civil capacity has been recognised at an early period in every system of law. In the Roman jurisprudence its consequences were very fully developed, and the provisions and terminology of that system have largely affected the subsequent legal treatment of the subject. Its leading principles were simple and well marked. The insane person having no intelligent will, and being thus incapable of consent, or voluntary action, could acquire no right and incur no responsibility by his own acts; his person and property were placed, after inquiry by the magistrate, under the control of a *curator*. The different terms by which the insane were known, such as *demens*, *furiosus*, *fatuus*, although no doubt signifying different types of insanity, did not infer any differences of legal treatment. They were popular names which were used somewhat differently, but which all denoted the complete deprivation of reason. During the Middle Ages the insane were but little protected, or regarded by law. Their legal acts were annulled, and their property placed under control, but little or no attempt was made to supervise their personal treatment. In England the wardship of idiots and lunatics, which was annexed before the reign of Edward II. to the King's prerogative, had regard chiefly to the control of their lands and estates, and was only gradually elaborated into the systematic control of their person and property now exercised in chancery. Those whose means were insignificant were left to the care of their relations, or to charity. In criminal law the plea of insanity was unavailing, except in extreme cases.

Legislators and jurists have done little more than merely to indicate some of the most obvious divisions of insanity, without undertaking anything like a systematic classification of its various forms, and the English common law originally recognised but two kinds of insanity, *idiocy* and *lunacy*, the subjects of which were designated by the term *non compos mentis*, which was used in a generic sense, and meant to embrace all who, from defect of understanding, require the protection of the law. An occasional attempt has been made by jurists to attach some definite ideas to these terms, and to point out the various descriptions of persons to whom they may be applied. Lord Coke says, there are four kinds of men who may be said to be *non compos mentis*:—(1) An idiot, who from his nativity, by a perpetual infirmity is *non compos*; (2) He that by sickness, grief, or other accident, wholly loseth his memory and understanding; (3) A lunatic that hath sometimes his understanding and sometimes not, *aliquando gaudet lucidis intervallis*; and therefore he is called *non compos mentis* so long as he hath not understanding; (4) He that by his own vicious act for a time depriveth himself of his memory and understanding, as that he is drunken (Coke's Littleton 247a).

Nothing can show more plainly how imperfect were the notions of the early law-writers concerning insanity than this classification of insane persons, and their attempts to define the several classes. An idiot is defined to be a person who cannot count or number twenty pence, or tell who was his father or mother, or how old he is, so as it may appear that he hath no understanding of reasons what shall be for his profit, or what shall be for his loss; but if he have sufficient understanding to know, and understand his letters, and to read by teaching or information, he is not an idiot. Now the truth is, that many of those whose idiocy is unquestionable, are capable of attaining the kind of knowledge herein specified, by means of the ordinary intercourse with men, or of special teaching. The entire loss of memory and understanding, attributed to this second class, is observed only as a sequel to madness or some other disease, or as the result of some powerful moral causes; so that if this is to be considered an essential character of madness, by much the larger proportion of madmen will be altogether excluded from this classification; for, instead of wholly losing their understanding, they are for the most part perfectly rational on some topics, and in some relations of life, and a little effort is frequently necessary in order to detect the fact of the understanding being at all impaired.

It by no means follows, that a person declared to be *non compos* by due process of law, is to be considered on that account merely to be irresponsible for his criminal acts. This is a question entirely distinct, and is determined upon very different views of the nature of insanity, and of its effects on the operations of the mind; and, here it is, that the lawyer encroaches most on the domain of the physician. The first attempt to point out precisely those conditions of insanity in which the civil and criminal responsibilities are unequally affected, was made by Lord Hale. "There is a partial insanity," says he "and a total insanity. The former is either in respect to things, *quoad hoc vel illud insanire.* Some persons that have a competent use of reason in respect of some subjects, are yet under a particular *dementia*, in respect of some particular discourses, subjects, or applications, or else it is partial in respect of degrees, and this is the condition of very many, especially melancholy persons, who for the most part discover their defect in excessive fears and griefs, and yet are not wholly destitute of the use of reason; and this partial insanity seems not to excuse them, in the committing of any offence for its matter capital; for, doubtless, most persons that are felons of themselves, and others are under a degree of partial insanity, when these commit these offences. It is very difficult to define the invisible line that divides perfect and partial insanity, but it must rest upon circumstances duly to be weighed and considered both by judge and jury, lest on the one side there be a kind of inhumanity towards the defects of human nature; or, on the other side, too great an indulgence given to great crimes." (Hale's Pleas of the Crown, 30.) So strongly was this celebrated jurist possessed with this idea, that it is this strength and capacity of the mind only that are affected by insanity, that he has actually founded upon it a test of criminal responsibility. "Such a person," says he, "as labouring under melancholy distempers, hath yet ordinarily as great understanding as ordinarily a child of fourteen years hath, is such a person as may be guilty of treason or felony." As if the only difference between sanity and insanity were precisely that which is made by difference of age, and as if there could be two things more unlike than the mind of a person "labouring under melancholy distempers," and that of a child fourteen years old.

The doctrines thus dogmatically laid down by Lord Hale, have exerted no inconsiderable influence on the judicial opinions of his successors; and his high authority has often been invoked against the plea of insanity, whenever it has been urged by the voice of philanthropy and true science. If, too, in consequence of the common tendency of indulgence in forced and unwarrantable constructions, whenever a point is to be gained, his principles have been made to mean far more than he ever designed. The fact impressively teaches the importance of clear and well-defined terms, in the expression of scientific truths, as well as of enlarged practical information, relative to the subjects to which they belong. In the time of this eminent jurist, insanity was a much less frequent disease than it now is, and the popular notions concerning it were derived from those wretched inmates of the madhouse, whom chains and stripes, cold and filth, had reduced to the stupidity of the idiot, or exasperated to the fury of a demon. Those nice shades of the disease in which the mind, without being wholly driven from its propriety, pertinaciously clings to some absurd delusion, were either regarded as something very different from real madness, or were too few, too far removed from the common gaze, and too soon converted by bad management into the more active forms of the disease to enter much into the general ideas entertained of madness. Could Lord Hale have contemplated the scenes presented by the lunatic asylums of our own times, we should undoubtedly have received from him a very different doctrine for the regulation of the decisions of after generations.

